# Lupeol and pristimerin do not inhibit activation of the human sperm CatSper Ca(2+)-channel

**DOI:** 10.12688/f1000research.109279.1

**Published:** 2022-02-24

**Authors:** Anders Rehfeld, Christian Marcus Pedersen

**Affiliations:** 1 Department of Drug Design and Pharmacology, Faculty of Health Sciences, University of Copenhagen, Copenhagen, 2200 Copenhagen N, Denmark; 2 Department of Biomedical Sciences, Faculty of Health Sciences, University of Copenhagen, Copenhagen, 2200 Copenhagen N, Denmark; 3 Department of Growth and Reproduction, University of Copenhagen, Rigshospitalet, Copenhagen, 2100 Copenhagen E, Denmark; 4 Department of Chemistry, University of Copenhagen, Copenhagen, 2200 Copenhagen N, Denmark

**Keywords:** Fertility, CatSper, Male reproduction, Lupeol, Pristimerin, Sperm function

## Abstract

Opposing findings have been published on the regulation of the sperm-specific Ca ^2+^ channel CatSper (cation channel of sperm) in human sperm cells by the plant triterpenoids lupeol and pristimerin. While the original study on this topic found these triterpenoids to act as potent inhibitors of human CatSper, subsequent studies have failed to replicate such an inhibitory effect. It has been suggested that these issues could in part be due to purity issues and/or batch variation between the plant-derived extracts of lupeol and pristimerin obtained for the studies. The aim of this study was to elucidate this controversy by investigating the batches of lupeol and pristimerin used in our previous study with state-of-the-art ^1^H-, ^13^C- and 2D-nuclear magnetic resonance (NMR) methods to reveal potential purity and/or batch variation issues. When comparing the NMR-spectra obtained from ^1^H-NMR and ^13^C-NMR with previously published NMR-spectra for lupeol and pristimerin, we could confirm that both the lupeol and pristimerin batch were ≥95 % pure. These results confirm the validity of the findings in our previous study for lupeol and pristimerin, showing that lupeol and pristimerin do not inhibit activation of CatSper in human sperm. In conclusion, using ^1^H-, ^13^C- and 2D-NMR methods, we confirm that the lupeol and pristimerin batches used in our previous study were ≥95 % pure and thereby fail to identify any purity issues and/or batch variation that could explain the observed inability of lupeol and pristimerin to inhibit activation of CatSper in human sperm.

The putative inhibitory action of the two plant triterpenoids lupeol and pristimerin on the activation of the human sperm CatSper Ca(2+)-channel has recently been debated in the scientific literature. The original study on this subject ( Mannowetz *et al.*, 2017) indicated that these triterpenoids act as very potent and efficacious inhibitors of progesterone-activated CatSper-currents in human sperm cells with IC _50_-values in the lower nM range, and a follow-up study by the same research group confirmed the inhibitory action of pristimerin on progesterone-induced Ca(2+)-influxes via CatSper through measurements in the principal piece of the flagellum in single human sperm cells ( Mannowetz *et al.*, 2018).

In contrast to these findings, two studies from independent research groups failed entirely to replicate any inhibitory action for neither lupeol nor pristimerin on progesterone-induced Ca(2+)-influxes through CatSper in populations of human sperm cells ( Brenker *et al.*, 2018; Rehfeld, 2020) and progesterone-activated CatSper-currents in single human sperm cells ( Brenker *et al.*, 2018), even when exposing the sperm cells to lupeol and pristimerin at much higher µM concentrations.

The complete failure of these studies to replicate the findings from ( Mannowetz *et al.*, 2017; Mannowetz *et al.*, 2018) is highly concerning since a patent has been filed ( Lishko & Mannowetz, 2018) and a company (YourChoice Therapeutics, CA, US) has been formed based on the original discovery by ( Mannowetz *et al.*, 2017; Mannowetz *et al.*, 2018) that lupeol and pristimerin act as potent inhibitors of human CatSper and could thus potentially be used as novel male and female contraceptives.

Since the publication of the most recent study on this matter ( Rehfeld, 2020), the corresponding author was contacted by researchers who questioned the validity of the results presented in the study for lupeol and pristimerin, i.e., the inability to reproduce the inhibitory action of these triterpenoids on human CatSper, and suggested that the failure to identify such an inhibitory effect on human CatSper could be due to purity issues and/or batch variation between the plant-derived extracts of lupeol and pristimerin obtained for the study from Cayman Chemicals (MI, USA).

Although Cayman Chemicals stated that the lupeol and pristimerin batches were delivered with a purity of ≥98 %, we fully agreed with these researchers that it would be good scientific conduct and of general interest of the field of human sperm physiology to examine the two stocks solutions used in ( Rehfeld, 2020), i.e., a 5 mM pristimerin dimethylsulfoxid (DMSO) stock and a 1 mM lupeol ethanol stock, using state-of-the-art ^1^H-, ^13^C- and 2D-nuclear magnetic resonance (NMR) methods (Bruker 500 MHz Ultrashield Plus equipped with a CryoProbe, Bruker, Germany) to reveal potential purity and/or batch variation issues in these stocks.

To prepare the stocks for the NMR-measurements, we first evaporated the ethanol from the lupeol stock, removed the DMSO from the pristimerin stock using an evaporation system (V-10 evaporator, Biotage, Sweden), and exchanged the solvent for both triterpenoids to deuterated chloroform (CDCl _3_). The raw NMR data can be found as *Underling* data ( Rehfeld, 2022a). When comparing the NMR-spectra obtained on the two stocks from ^1^H-NMR and especially ^13^C-NMR (see *Extended data* ( Rehfeld, 2022b)) with previously published NMR-spectra for lupeol and pristimerin ( Espindola *et al.*, 2018; Shwe *et al.*, 2019), we could confirm that Cayman Chemicals had indeed provided us with batches containing lupeol and pristimerin, respectively. Furthermore, the NMR-data showed that both lupeol and pristimerin were ≥95 % pure ( *Extended data* ( Rehfeld, 2022b)), despite the prolonged storage at -20 °C since conducting the experiments for ( Rehfeld, 2020).

Taken together, the results provided here confirms the validity of the findings in our previous study for lupeol and pristimerin ( Rehfeld, 2020), i.e., that the two plant triterpenoids lupeol and pristimerin do not inhibit activation of CatSper in human sperm. The findings in ( Rehfeld, 2020) are therefore still in line with the observations by ( Brenker *et al.*, 2018) and still contradicting the putative inhibitory action of lupeol and pristimerin on human CatSper described in ( Mannowetz *et al.*, 2017; Mannowetz *et al.*, 2018).

In conclusion, using state-of-the-art ^1^H-, ^13^C- and 2D-NMR methods, we confirm here that the lupeol and pristimerin stocks used in ( Rehfeld, 2020) were ≥95 % pure and thereby fail to identify any purity issues and/or batch variation that could explain the observed inability of these triterpenoids to inhibit activation of CatSper in human sperm.

## Data availability

### Underlying data

Figshare. Raw ^1^H-, ^13^C- and 2D-NMR data for lupeol and pristimerin in MestReNova (Mnova) format. https://doi.org/10.6084/m9.figshare.19181087.v1 ( Rehfeld, 2022a).

Data are available under the terms of the Creative Commons Zero "No rights reserved" data waiver (CC0 1.0 Public domain dedication).

Raw ^1^H-, ^13^C- and 2D-NMR data for lupeol and pristimerin in MestReNova (Mnova) format are also available at the BMRbig repository, part of the Biological Magnetic Resonance Data Bank (BMRB), with ID: BMRbig35, https://bmrbig.org/released/bmrbig35.

### Extended data

Figshare: Supplementary file 1. https://doi.org/10.6084/m9.figshare.19134488.v1 ( Rehfeld, 2022b).

Data are available under the terms of the Creative Commons Attribution 4.0 International license (CC-BY 4.0).
