# Lupeol and pristimerin do not inhibit activation of the human sperm CatSper Ca(2+)-channel

**DOI:** 10.12688/f1000research.109279.2

**Published:** 2022-08-02

**Authors:** Anders Rehfeld, Christian Marcus Pedersen

**Affiliations:** 1 Department of Drug Design and Pharmacology, Faculty of Health Sciences, University of Copenhagen, Copenhagen, 2200 Copenhagen N, Denmark; 2 Department of Biomedical Sciences, Faculty of Health Sciences, University of Copenhagen, Copenhagen, 2200 Copenhagen N, Denmark; 3 Department of Growth and Reproduction, University of Copenhagen, Rigshospitalet, Copenhagen, 2100 Copenhagen E, Denmark; 4 Department of Chemistry, University of Copenhagen, Copenhagen, 2200 Copenhagen N, Denmark

**Keywords:** Fertility, CatSper, Male reproduction, Lupeol, Pristimerin, Sperm function

## Abstract

Opposing findings have been published on the regulation of the sperm-specific Ca ^2+^ channel CatSper (cation channel of sperm) in human sperm cells by the plant triterpenoids lupeol and pristimerin. While the original study on this topic found these triterpenoids to act as potent inhibitors of human CatSper, subsequent studies have failed to replicate such an inhibitory effect. It has been suggested that these issues could in part be due to purity issues and/or batch variation between the plant-derived extracts of lupeol and pristimerin obtained for the studies. The aim of this study was to elucidate this controversy by investigating the batches of lupeol and pristimerin used in our previous study with state-of-the-art ^1^H-, ^13^C- and 2D-nuclear magnetic resonance (NMR) methods to reveal potential purity and/or batch variation issues. When comparing the NMR-spectra obtained from ^1^H-NMR and ^13^C-NMR with previously published NMR-spectra for lupeol and pristimerin, we could confirm that both the lupeol and pristimerin batch were ≥95 % pure. These results confirm the validity of the findings in our previous study for lupeol and pristimerin, showing that lupeol and pristimerin do not inhibit activation of CatSper in human sperm. In conclusion, using ^1^H-, ^13^C- and 2D-NMR methods, we confirm that the lupeol and pristimerin batches used in our previous study were ≥95 % pure and thereby fail to identify any purity issues and/or batch variation that could explain the observed inability of lupeol and pristimerin to inhibit activation of CatSper in human sperm.

The putative inhibitory action of the two plant triterpenoids lupeol and pristimerin on the activation of the human sperm CatSper Ca(2+)-channel has recently been debated in the scientific literature. The original study on this subject ( Mannowetz *et al.*, 2017) indicated that these triterpenoids act as very potent and efficacious inhibitors of progesterone-activated CatSper-currents in human sperm cells with IC _50_-values in the lower nM range, and a follow-up study by the same research group confirmed the inhibitory action of pristimerin on progesterone-induced Ca(2+)-influxes via CatSper through measurements in the principal piece of the flagellum in single human sperm cells ( Mannowetz *et al.*, 2018).

In contrast to these findings, two studies from independent research groups failed entirely to replicate any inhibitory action for neither lupeol nor pristimerin on progesterone-induced Ca(2+)-influxes through CatSper in populations of human sperm cells ( Brenker *et al.*, 2018b; Rehfeld, 2020) and progesterone-activated CatSper-currents in single human sperm cells ( Brenker *et al.*, 2018b), even when exposing the sperm cells to lupeol and pristimerin at much higher µM concentrations.

The complete failure of these studies to replicate the findings from ( Mannowetz *et al.*, 2017; Mannowetz *et al.*, 2018) is highly concerning since a patent has been filed ( Lishko & Mannowetz, 2018) and a company (YourChoice Therapeutics, CA, US) has been formed based on the original discovery by ( Mannowetz *et al.*, 2017; Mannowetz *et al.*, 2018) that lupeol and pristimerin act as potent inhibitors of human CatSper and could thus potentially be used as novel male and female contraceptives.

Since the publication of the most recent study on this matter ( Rehfeld, 2020), the corresponding author was contacted by researchers who questioned the validity of the results presented in the study for lupeol and pristimerin, i.e., the inability to reproduce the inhibitory action of these triterpenoids on human CatSper, and suggested that the failure to identify such an inhibitory effect on human CatSper could be due to purity issues and/or batch variation between the plant-derived extracts of lupeol and pristimerin obtained for the study from Cayman Chemicals (MI, USA).

Although Cayman Chemicals stated that the lupeol and pristimerin batches were delivered with a purity of ≥98 %, we fully agreed with these researchers that it would be good scientific conduct and of general interest of the field of human sperm physiology to examine the two stocks solutions used in ( Rehfeld, 2020), i.e., a 5 mM pristimerin dimethylsulfoxid (DMSO) stock and a 1 mM lupeol ethanol stock, using state-of-the-art ^1^H-, ^13^C- and 2D-nuclear magnetic resonance (NMR) methods (Bruker 500 MHz Ultrashield Plus equipped with a CryoProbe, Bruker, Germany) to reveal potential purity and/or batch variation issues in these stocks.

To prepare the stocks for the NMR-measurements, we first evaporated the ethanol from the lupeol stock, removed the DMSO from the pristimerin stock using an evaporation system (V-10 evaporator, Biotage, Sweden), and exchanged the solvent for both triterpenoids to deuterated chloroform (CDCl _3_). The raw NMR data can be found as *Underling* data ( Rehfeld, 2022a). When comparing the NMR-spectra obtained on the two stocks from ^1^H-NMR and especially ^13^C-NMR (see *Extended data* ( Rehfeld, 2022b)) with previously published NMR-spectra for lupeol and pristimerin ( Espindola *et al.*, 2018; Shwe *et al.*, 2019), we could confirm that Cayman Chemicals had indeed provided us with batches containing lupeol and pristimerin, respectively. Furthermore, the NMR-data showed that both lupeol and pristimerin were ≥95 % pure ( *Extended data* ( Rehfeld, 2022b)), despite the prolonged storage at -20 °C since conducting the experiments for ( Rehfeld, 2020).

Taken together, the results provided here confirms the validity of the findings in our previous study for lupeol and pristimerin ( Rehfeld, 2020), i.e., that the two plant triterpenoids lupeol and pristimerin do not inhibit activation of CatSper in human sperm. The findings in ( Rehfeld, 2020) are therefore still in line with the observations by ( Brenker *et al.*, 2018b) and still contradicting the putative inhibitory action of lupeol and pristimerin on human CatSper described in ( Mannowetz *et al.*, 2017; Mannowetz *et al.*, 2018).

The data presented here do not explain the discrepancy between the results by ( Mannowetz *et al.*, 2017; Mannowetz *et al.*, 2018) and ( Brenker *et al.*, 2018b; Rehfeld, 2020). In their follow-up study ( Mannowetz *et al.*, 2018) suggested that the irreproducibility of their findings was due to two issues: 1) Differences between electrophysiological protocols. It was claimed that ( Brenker *et al.*, 2018b) used an electrical driving force of only 20 mV (generated by stepping from a holding potential of −80 mV to −100 mV) to activate CatSper currents. This was argued to be too small to reliably assess inward CatSper currents under conditions in which the bath and pipette solutions contain equal concentrations of the major permeant ion, i.e., in the absence of a chemical driving force. 2) Differences in the Ca(2+)-imaging assays used to measure Ca(2+)-influxes in human sperm cells. ( Rehfeld, 2020) and ( Brenker *et al.*, 2018b) measured Ca(2+)-influxes in populations of human sperm, whereas ( Mannowetz *et al.*, 2018) measured Ca(2+)-influxes in the principal piece of the flagellum using single-cell imaging. ( Mannowetz *et al.*, 2018) claimed that CatSper-mediated Ca(2+)-influxes must be recorded specifically in the principal piece of the flagellum in order to avoid interference from strong fluorescent signals from the head, which could mask the fluorescence changes in the flagellum. These two arguments put forward by ( Mannowetz *et al.*, 2018) are discussed below.

First, the experimental protocol used by ( Brenker *et al.*, 2018) was also used by this group in two recent publications ( Brenker *et al.*, 2018a; Schiffer *et al.*, 2020) to record CatSper-currents from human sperm. Recordings from CatSper-deficient sperm by ( Schiffer *et al.*, 2020) demonstrate that the currents recorded under these conditions are indeed carried by CatSper. In contrast to the cesium-based bath and pipette solutions used by ( Mannowetz *et al.*, 2017), ( Brenker *et al.*, 2018b) used a sodium-based divalent-free (NaDVF) bath solution together with a cesium-based pipette solution. This means that inward CatSper currents are carried by Na ^+^ and outward currents by Cs ^+^. Under these specific conditions the reversal potential (V _rev_) for monovalent CatSper currents is ~30 mV ( Brenker *et al.*, 2018a; Schiffer *et al.*, 2020). The driving force (V _DF_) for monovalent CatSper currents at a certain membrane potential (V _m_) is given by V _DF_ = (V _m_ – V _rev_). Accordingly, at a membrane potential of −100 mV, the driving force is ~130 mV rather than 20 mV as suggested by ( Mannowetz *et al.*, 2018), why sizeable inward currents via CatSper can be recorded at this membrane potential using the experimental protocol in ( Brenker *et al.*, 2018b). Thus, the argument put forward by ( Mannowetz *et al.*, 2018) is based on a biophysical misconception. Moreover, Figure 2D in ( Brenker *et al.*, 2018b) demonstrates the failure of pristimerin and lupeol to inhibit resting and progesterone-activated CatSper currents across the entire range of membrane potentials tested and not just at −100 mV as shown in Figure 2E in ( Brenker *et al.*, 2018b). Taken together, the claim by ( Mannowetz *et al.*, 2018) that the irreproducibility of their results is due to differences in the electrophysiological protocols seems invalid.

Secondly, concerning the differences in Ca(2+)-imaging protocols. Already in 1996, Ca(2+)-imaging in populations of human sperm was used to identify potent antagonists of the progesterone-induced Ca(2+)-influxes in human sperm cells ( Blackmore *et al.*, 1996). In ( Rehfeld, 2020) the sperm population based Ca(2+)-imaging assay confirmed the inhibitory action of the specific CatSper inhibitor RU1968 ( Rennhack *et al.*, 2018), but completely failed to identify any inhibition of progesterone-induced Ca(2+)-influxes by lupeol and pristimerin in experiments testing these compounds side-by-side, see Figure 6 in ( Rehfeld, 2020). This fails to support the claim by ( Mannowetz *et al.*, 2018) that the irreproducibility of their results is due to differences in Ca(2+)-imaging protocols.

In a recent review article on natural products with a potential for nonhormonal male contraception ( Shunnarah *et al.*, 2021) the authors discuss the controversy regarding the action of lupeol and pristimerin on human CatSper and state the following: “ *These contradictory findings suggest a need for further studies to confirm the action or lack of action of the plant triterpenoids on the CatSper channel. The conflicting results also demonstrate the difficulties in reproducibility of results, which is often a barrier to studies of natural compounds in general.*”. We fully agree with this statement and believe that our study follows up on this suggestion. We encourage other researchers to test lupeol and pristimerin for actions on human CatSper in their own lab and offer them the opportunity to send us their batches of lupeol and pristimerin for state-of-the-art ^1^H-, ^13^C- and 2D-NMR analyses. Also, we have contacted the research groups of behind the implicated studies on this controversy ( Brenker *et al.*, 2018b) and ( Mannowetz *et al.*, 2017; Mannowetz *et al.*, 2018) with this opportunity.

In conclusion, using state-of-the-art ^1^H-, ^13^C- and 2D-NMR methods, we confirm here that the lupeol and pristimerin stocks used in ( Rehfeld, 2020) were ≥95 % pure and thereby fail to identify any purity issues and/or batch variation that could explain the observed inability of these triterpenoids to inhibit activation of CatSper in human sperm.

## Data availability

### Underlying data

Figshare. Raw ^1^H-, ^13^C- and 2D-NMR data for lupeol and pristimerin in MestReNova (Mnova) format. https://doi.org/10.6084/m9.figshare.19181087.v1 ( Rehfeld, 2022a).

Data are available under the terms of the Creative Commons Zero "No rights reserved" data waiver (CC0 1.0 Public domain dedication).

Raw ^1^H-, ^13^C- and 2D-NMR data for lupeol and pristimerin in MestReNova (Mnova) format are also available at the BMRbig repository, part of the Biological Magnetic Resonance Data Bank (BMRB), with ID: BMRbig35, https://bmrbig.org/released/bmrbig35.

### Extended data

Figshare: Supplementary file 1. https://doi.org/10.6084/m9.figshare.19134488.v1 ( Rehfeld, 2022b).

Data are available under the terms of the Creative Commons Attribution 4.0 International license (CC-BY 4.0).
