# Supplementary material for: Lupeol and pristimerin do not inhibit activation of the human sperm CatSper Ca(2+)-channel
Source: F1000Res. 2022 Aug 2;11:222. Originally published 2022 Feb 24. [Version 2] doi: 10.12688/f1000research.109279.2 (PMC9363974; doi:10.12688/f1000research.109279.2)
Supplement: Peer review history

# Peer review: Reviewer response for version 2

**DOI:** 10.5256/f1000research.135864.r146345

**Published:** 2022-08-22

**Authors:** Tao Luo

**Affiliations:** 1 Institute of Life Science, School of Life Sciences, Nanchang University, Nanchang, China

The authors have made a comprehensive revision according to my issue and addressed all my issue. Now, I have no additional comments. I think the manuscript is suitable for indexing at this version.

Are arguments sufficiently supported by evidence from the published literature or by new data and results?

Yes

Is the conclusion balanced and justified on the basis of the presented arguments?

Yes

Is the rationale for commenting on the previous publication clearly described?

Yes

Are any opinions stated well-argued, clear and cogent?

Yes

Reviewer Expertise:

Male infertility

I confirm that I have read this submission and believe that I have an appropriate level of expertise to confirm that it is of an acceptable scientific standard.

# Peer review: Reviewer response for version 2

**DOI:** 10.5256/f1000research.135864.r146343

**Published:** 2022-08-09

**Authors:** João Ramalho-Santos

**Affiliations:** 1 CNC-Center for Neuroscience and Cell Biology, University of Coimbra, Coimbra, Portugal; 2 DCV-Department of Life Sciences, University of Coimbra, Coimbra, Portugal; 3 CIBB-Center for Innovative Biomedicine and Biotechnology, University of Coimbra, Coimbra, Portugal

I understand the authors' response in terms of the context it provided to my initial comments/reservations. I have no further comments.

Are arguments sufficiently supported by evidence from the published literature or by new data and results?

Partly

Is the conclusion balanced and justified on the basis of the presented arguments?

Partly

Is the rationale for commenting on the previous publication clearly described?

Partly

Are any opinions stated well-argued, clear and cogent?

Yes

Reviewer Expertise:

Sperm physiology, male and female infertility, reproductive and stem cell biology.

I confirm that I have read this submission and believe that I have an appropriate level of expertise to confirm that it is of an acceptable scientific standard.

# Peer review: Reviewer response for version 1

**DOI:** 10.5256/f1000research.120762.r129827

**Published:** 2022-05-04

**Authors:** Huafeng Wang

**Affiliations:** 1 Department of Cellular and Molecular Physiology, Yale School of Medicine, New Haven, CT, USA

The effects of lupeol and pristimerin on human CatSper have aroused a controversy since two PNAS papers were published from different groups (Mannowetz et al., 2017 and Brenker et al., 2018). This manuscript was aiming to uncover a potential possibility for a better explanation by examining the purity of lupeol and pristimerin, together with their previous work (Molecular Human Reproduction, 2020). It is believed the data in this manuscript was solid and convincing. However, is the lupeol and pristimerin tested in this manuscript from same batch with the other two groups? If not, it would be great to test those from the two groups individually, even though the purity was not mentioned in this controversy (Mannowetz et al., 2018 and Brenker et al., 2018).

Are arguments sufficiently supported by evidence from the published literature or by new data and results?

Partly

Is the conclusion balanced and justified on the basis of the presented arguments?

Partly

Is the rationale for commenting on the previous publication clearly described?

Yes

Are any opinions stated well-argued, clear and cogent?

Partly

Reviewer Expertise:

sperm, CatSper, ion channel, electrophysiology

I confirm that I have read this submission and believe that I have an appropriate level of expertise to confirm that it is of an acceptable scientific standard, however I have significant reservations, as outlined above.

# Response

**Published:** 2022-07-05

**Authors:** Anders Rehfeld

**Affiliations:** University of Copenhagen, Denmark

We have now contacted these groups to offer them NMR-analyses of their lupeol and pristimerin batches.

# Peer review: Reviewer response for version 1

**DOI:** 10.5256/f1000research.120762.r129824

**Published:** 2022-04-13

**Authors:** Tao Luo

**Affiliations:** 1 Institute of Life Science, School of Life Sciences, Nanchang University, Nanchang, China

Different research groups showed contradictory results on whether plant triterpenoids, lupeol and pristimerin, inhibit the activation of CatSper. It has been suggested that these issues could in part be due to purity issues and/or batch variation between the plant-derived extracts of lupeol and pristimerin obtained for the studies.

 This manuscript aimed to elucidate this controversy by investigating the batches of lupeol and pristimerin used in their previous study using state-of-the-art 1H-, 13C- and 2D-nuclear magnetic resonance (NMR) methods. The authors confirm that the lupeol and pristimerin batches used in their previous study were ≥95 % pure.  Therefore, they concluded that the purity issues and/or batch variation could not explain the inability of lupeol and pristimerin to inhibit activation of CatSper in human sperm found in their previous study. The opinions stated are well-argued, clear and cogent. The arguments were sufficiently supported by evidence from the new data.

 However, the manuscript did not discuss or respond to the other two important issues argued by Mannowetz et al. 2018:

1. An electrical driving force of 20 mV generated from a holding potential of −80 to −100 mV, as shown by Brenker et al. 2018, is neither enough to reliably assess inward CatSper currents nor to estimate “fold current increase”.
2. If it is of interest to study CatSper-mediated calcium influx into spermatozoa, individual principle pieces (PPs) as regions of interest must be analyzed.

If these issues are addressed, the controversy may be better elucidated.

Are arguments sufficiently supported by evidence from the published literature or by new data and results?

Yes

Is the conclusion balanced and justified on the basis of the presented arguments?

Yes

Is the rationale for commenting on the previous publication clearly described?

Yes

Are any opinions stated well-argued, clear and cogent?

Yes

Reviewer Expertise:

Male infertility

I confirm that I have read this submission and believe that I have an appropriate level of expertise to confirm that it is of an acceptable scientific standard, however I have significant reservations, as outlined above.

# Response

**Published:** 2022-07-05

**Authors:** Anders Rehfeld

**Affiliations:** University of Copenhagen, Denmark

We have now addressed these two issues in the discussion of the updated manuscript.

# Peer review: Reviewer response for version 1

**DOI:** 10.5256/f1000research.120762.r125363

**Published:** 2022-03-02

**Authors:** João Ramalho-Santos

**Affiliations:** 1 CNC-Center for Neuroscience and Cell Biology, University of Coimbra, Coimbra, Portugal; 2 DCV-Department of Life Sciences, University of Coimbra, Coimbra, Portugal; 3 CIBB-Center for Innovative Biomedicine and Biotechnology, University of Coimbra, Coimbra, Portugal

My comments have to do with the fact that, while the topic is very worthwhile, this is not exactly (in my view) a straightforward Correspondence. The steps are as follows:

1. Originally a paper is published in Proceedings of the National Academy of Sciences of the United States of America (PNAS) in 2017 showing that Lupeol and pristimerin strongly inhibits the CatSper channel, the implication being that they could be used in male contraception, and apparently a company was formed with that in mind.
2. In 2018 (also in PNAS) other authors question this data suggesting that there are, in fact, no effects.
3. The original 2017 authors contest this, stand by their results and suggest that their data is valid and that the negative results are based on monitoring different things. Sample purity is not mentioned in this exchange.
4. It should be noted that none of the authors of this correspondence were involved in this controversy, the first author did work with the team that published the 2018 paper, and confirmed the absence of results with CatSper not being inhibited by Lupeol and pristimerin in a 2020 Molecular Human Reproduction (MHR) paper.

In this correspondence the authors perform NMR spectra to show that Lupeol and pristimerin are as pure as possible, and that the absence of inhibition of CatSper is therefore a true result. One authors is an expert on sperm (and CatSper in particular), the other on chemical structures, so I have no issues with the data here at all. However, I doubt that this will in any meaningful way solve the contradiction. Using other batches of Lupeol and pristimerin might be a possibility, or at least framing the discussion a bit more thoroughly and discussing/updating the issues raised in the 2018 exchange, that, as stated, never mention sample purity. But I do respect the authors not wanting to do this, as they were not part of those papers.

Are arguments sufficiently supported by evidence from the published literature or by new data and results?

Partly

Is the conclusion balanced and justified on the basis of the presented arguments?

Partly

Is the rationale for commenting on the previous publication clearly described?

Partly

Are any opinions stated well-argued, clear and cogent?

Yes

Reviewer Expertise:

Sperm physiology, male and female infertility, reproductive and stem cell biology.

I confirm that I have read this submission and believe that I have an appropriate level of expertise to confirm that it is of an acceptable scientific standard, however I have significant reservations, as outlined above.

# Response

**Published:** 2022-03-03

**Authors:** Anders Rehfeld

**Affiliations:** University of Copenhagen, Denmark

I fully agree with the reviewer in his comment that this is not a straightforward Correspondence. I would have preferred to be able to publish all my data on this issue as a Letter to the original PNAS paper, but unfortunately, there was a 6-month limit to submit such Letters to articles in PNAS and I did not have any data ready within that timeframe. My way into writing this Correspondence was this:

  

1. I read the original 2017 PNAS paper and was very excited about the novel CatSper-inhibitor compounds Lupeol and Pristimerin, which seemed to be highly potent and efficacious compared to other known CatSper-inhibitors. I therefore ordered these compounds to use them in my own studies as CatSper-inhibitors.
2. I received Lupeol and Pristimerin, diluted them according to the recommendation of the vendor and started testing them in my own experimental setup. However, I could not identify any inhibitory effects of the compounds on CatSper in human sperm cells.
3. Puzzled, I contacted the authors of the original 2017 PNAS paper to discuss what I might have done wrong. The authors of the original 2017 PNAS paper suggested me to test Lupeol and Pristimerin prepared at different stock concentrations in different solvents, with or without sonication, as well as from different vendors. However, in the end I could still not observe any inhibition of human CatSper in my assay.
4. At this point I decided to re-examine both the two triterpenoids, Lupeol and Pristimerin, and the steroids investigated in the original 2017 PNAS paper using my own experimental setup. This collected work is what I published in the 2020 Molecular Human Reproduction paper.
5. After publishing this 2020 Molecular Human Reproduction paper, I was contacted per e-mail by researchers who questioned the validity of the results presented in the study for Lupeol and Pristimerin, and suggested that my failure to identify inhibitory effects on human CatSper could be due to purity issues and/or batch variation between the plant-derived extracts of Lupeol and Pristimerin obtained for the study. As the reviewer states, this purity issue was not mentioned in the 2018 PNAS exchange.
6. I had to agree with the researchers that this could of course be true and something that I should have investigated before submitting the 2020 Molecular Human Reproduction paper. I thus contacted, Christian Marcus Pedersen, a chemical scientist from the chemical department at the University of Copenhagen, and asked him to perform nuclear magnetic resonance analyses of the Lupeol and Pristimerin stocks I used for the 2020 Molecular Human Reproduction study.
7. Christian Marcus Pedersen performed nuclear magnetic resonance analyses of my Lupeol and Pristimerin batches and thankfully both compounds were present at high purity, even despite the prolonged storage at -20 °C since conducting the experiments for the 2020 Molecular Human Reproduction paper.
8. At this point I simply wanted to share these additional important data, which I thought were highly relevant for the discussion of the putative inhibitory effects of Lupeol and Pristimerin on human CatSper. Unfortunately, it was not possible for me to simply add these nuclear magnetic resonance data to the 2020 Molecular Human Reproduction paper, why I had to identify another journal to get the data published, accessible, and citable to other researchers of the human sperm physiology field. Luckily Correspondence articles at F1000 Research were suitable for exactly this.

Of note, I believe that I discussed several issues raised in the 2018 PNAS exchange quite thoroughly in the 2020 Molecular Human Reproduction paper, e.g., the claim that Ca ^2+^-signals must be recorded specifically from the principal piece of the flagellum only to be able to observe an inhibitory effect on CatSper in human sperm cells, which did not agree with my observations using the other CatSper-inhibitor RU1968. However, if other reviewers also suggest this, we can of course update our Correspondence to discuss the issues raised in the 2018 PNAS exchange more thoroughly.

 I fully agree with the reviewer that this Correspondence may not solve this controversy. However, in a recent review article *“Natural Products with Potential for Nonhormonal Male Contraception”* (J. Nat. Prod. 2021, 84, 2762−2774), they also discuss the controversy regarding the action of Lupeol and Pristimerin on human CatSper, and state the following: *“These contradictory findings suggest a need for further studies to confirm the action or lack of action of the plant triterpenoids on the CatSper channel. The conflicting results also demonstrate the difficulties in reproducibility of results, which is often a barrier to studies of natural compounds in general.”*.

 I believe that we are following up on this exact suggestion with this Correspondence and I hope it will be helpful for solving the issue and reaching consensus in the human sperm physiology field at some point.

 In the end, my hope is that other researchers reading our Correspondence will obtain Lupeol and Pristimerin themselves and test these compounds for effects on CatSper in human sperm cells in their own lab and hopefully at some point publish their findings like we have done here. This will be the only way to solve this controversy in my opinion.

# Response

**Published:** 2022-07-05

**Authors:** Anders Rehfeld

**Affiliations:** University of Copenhagen, Denmark

As the other reviewers also suggest that we discuss the issues raised in the 2018 PNAS exchange, we have now update our Correspondence manuscript to address these issues more thoroughly.
